# Machine Learning-Driven Computer Vision System for Automated Fat and Energy Quantification in Human Milk Microcapillaries

**DOI:** 10.3390/s26061756

**Published:** 2026-03-10

**Authors:** Lujan E. Huamanga-Chumbes, Erwin J. Sacoto-Cabrera, Jaime Lloret, Vinie Lee Silva-Alvarado, Alfz Huicho-Mendigure, Edison Moreno-Cardenas

**Affiliations:** 1 TESLA Laboratory, Universidad Nacional de San Antonio Abad del Cusco (UNSAAC), Cusco 08003, Peru; 201250@unsaac.edu.pe (L.E.H.-C.); alfz.huicho@unsaac.edu.pe (A.H.-M.); 2 GIHP4C, Universidad Politécnica Salesiana, Cuenca 010102, Ecuador; esacoto@ups.edu.ec; 3 Instituto de Investigación para la Gestión Integrada de Zonas Costeras, Universitat Politècnica de València, Carrer del Paranimf 1, Grao de Gandia, 46730 Valencia, Spain; jlloret@dcom.upv.es (J.L.); vlsilalv@doctor.upv.es (V.L.S.-A.); 4 Technology and Engineering Group, EM Research & Tech, Cusco 08003, Peru

**Keywords:** human milk, computer vision system, machine learning, neonatal nutrition, image segmentation, uncertainty analysis, clinical informatics

## Abstract

Neonatal health requires precise lipid quantification in human milk to ensure proper nutritional development. Traditional manual methods, such as the creamatocrit, are limited by human-induced bias and significant measurement uncertainty. This study presents a low-cost Computer Vision System acting as an automated optical sensing modality for estimate the cream fraction (*c*) using advanced Machine Learning regression, which is subsequently used to derive fat and energy quantification through established analytical equations. The system is optimized for the Gold-LED spectrum, which enhances the dynamic range to 226 a.u. for robust feature extraction. We evaluated 28 distinct ML regression models across three feature spaces (Gray Scale, RGB, and Combined). The results, based on 6400 samples, demonstrate that the Rational Quadratic GPR model achieved the highest predictive stability with a coefficient of determination of $R^{2}=0.867$. This computational framework achieved a 57.5% reduction in relative error compared to manual benchmarks. SHAP analysis indicates that the model selectively attributes higher importance to Red channel intensities and Blue contrast gradients, which correspond to the optical scattering characteristics of lipid globules. These findings validate the system as a stable sensing modality for non-invasive quantification. The proposed architecture integrates cost-effective hardware with high-precision analytical modeling, offering a reagent-free and operationally feasible alternative for standardized nutritional assessment in neonatal intensive care units and milk banks.

## 1. Introduction

One of the most essential pillars for the immediate survival of newborns and their long-term neurocognitive and metabolic development is neonatal nutrition. Within the ecosystem of Neonatal Intensive Care Units (NICU), human milk (HM) is recognized as the nutritional gold standard in milk banks (MB) [1,2]. However, its nature is not static; it constitutes a highly dynamic biological matrix linked to a biological signature that reflects maternal factors, gestational age, and circadian rhythms [3,4]. Within this complex matrix, fatty acids (FA) are critical, contributing with neutral lipids and representing the most energy-dense component necessary for neurological and retinal maturation [1,3,5].

The central problem is that lipid content is the most volatile macronutrient in HM, with fluctuations that can compromise postnatal growth trajectories if not accurately quantified prior to administration [6,7]. Recent studies emphasize that the specific fatty acid (FA) profile undergoes critical changes during milk maturation and in response to maternal metabolic status [5]. Factors such as pre-pregnancy body mass index (pBMI) and dietary intake of long-chain polyunsaturated fatty acids (LCPUFA) significantly alter the quality of the milk product [8], leading to essential nutrient deficits in very-low-birth-weight infants [9]. Given this heterogeneity, the practice of using breast milk without precise quantification is insufficient to meet the requirements of preterm neonates who depend on personalized supplementation to avoid extrauterine growth restriction (EUGR) [10,11].

The management of this variability is exacerbated by technical limitations at the point of care. Effective monitoring of caloric density is mandatory [12], but current tools are often costly and inaccessible to many health centers. In particular, there are no devices developed specifically for measuring human breast milk [13], suggesting a significant lack of solutions focused on women’s health needs. Reference methods like infrared spectroscopy [14] need specialized maintenance and large capital expenditures, which are not always accessible in rural areas. Additionally, because components like lipids and insulin vary significantly between feeding phases, the absence of standardized sampling protocols introduces bias [15]. Thus, it is critical to create quick, portable, and easily accessible solutions that optimize the alignment between clinical viability and laboratory standards [16]. In response to this need, computer vision systems (CVS) function as non-invasive optical sensors that translate spatial and intensity data into compositional metrics. This approach provides a high-resolution alternative to manual inspection by utilizing digital image processing as a primary sensing modality [17,18,19,20]. The physical principle of this sensing modality relies on the optical contrast at the interface of milk phases. The lipid-rich cream layer exhibits higher light-scattering and reflection coefficients compared to the aqueous serum, resulting in a distinct pixel intensity distribution. These distribution profiles encode the volumetric information of the sample, making quantitative regression feasible by mapping digital features to physical lipid concentration. The rise of telemedicine and the use of AI to predict complications, such as EUGR [21], demonstrate the viability of digital solutions to optimize neonatal care [22]. In this context, the combination of a minimalist hardware design with robust processing algorithms allows for superior traceability and quality control [23]. Nutrition personalization, supported by digital tools, facilitates the adaptation of nutritional strategies to the individual needs of the most fragile users [24]. Even with aggressive care bundles that include parenteral lipid emulsions to mitigate weight loss, the transition to optimal enteral nutrition remains a critical challenge [25,26]. The development of a system capable of isolating and quantifying the cream interface in microcapillaries in an automated manner not only eliminates the subjectivity of the analog creamatocrit method but also provides a scalable and economical tool.

On the other hand, recent advances in intelligent detection systems enable the integration of low-cost hardware with data-driven algorithms to perform reliable physical measurements in non-laboratory environments [27]. AI-enhanced integrated control platforms have demonstrated stable real-world implementation of sensor-based systems. Likewise, machine learning (ML) [28,29] applied to physical sensor data has enabled the automation of diagnostic tasks that replace subjective human assessment [20]. The combination of image processing with IoT-based acquisition architectures further confirms computer vision as a scalable detection modality in real-world settings [30]. These advances reinforce the feasibility of objective, automated nutritional assessment through intelligent visual detection.

The main objective of this study is twofold. First, to automate the quantification of cream fraction using a low-cost computer vision system, eliminating the subjectivity inherent in the analogue creamatocrit method. Second, to validate a measurement model capable of operating with high fidelity under controlled lighting conditions. This is achieved by integrating a standardized acquisition prototype with microcapillaries, which allows the isolation and quantification of the cream interface. A central aspect of this approach is the implementation of a Rational Quadratic Gaussian Process Regression (GPR) model. This specific ML architecture was selected after a thorough evaluation of 28 different regression algorithms due to its superior ability to model the complex non-linear relationship between pixel-based volumetric measurements and actual lipid concentration, while effectively managing the heteroscedasticity of biological samples. This system provides a robust, economical, scalable, and high-quality tool.

The rest of the study is divided into six additional sections. Section 2 details the most relevant related studies. Section 3 details the Materials and Methods. The results are presented in Section 4, followed by the Discussion in Section 5 and the Conclusions with future perspectives in Section 6.

## 2. Related Works

The evaluation of the nutritional quality of HM has historically relied on complex laboratory analyses [14]. These have required high-cost equipment, highly specialized personnel, and prolonged processing times that prevent immediate clinical decision-making [23]. Recent studies have underlined that the price of Mid-Infrared (MIR) instruments continues to increase due to their optical complexity and that alternative methods, such as ultrasound, although more economical, demand excessive sample volumes and are susceptible to calibration errors [6]. Beyond traditional analytical chemistry, the emergence of vision-as-sensor frameworks has redefined imaging pipelines as quantitative optical transducers. In these systems, the camera serves as a non-contact probe that captures the interaction between light and the biological sample. Unlike qualitative imaging, this sensing modality relies on the metrological extraction of physical boundaries and optical density variations, where the digital sensor replaces physical gauges to provide higher spatial resolution and repeatability. Given this scenario, computer vision has emerged as a disruptive solution, enabling non-destructive analysis that simulates human inspection capacity while offering superior mathematical objectivity.

At the most advanced level of this technological transition, Deep Learning (DL) and Convolutional Neural Networks (CNN) have demonstrated the ability to transform visual features into predictive metrics with unprecedented accuracy. Investigations by Dahiya et al. [31] have validated the use of models such as InceptionV3 and Vision Transformers to predict yield and lacteal nutritional quality from images, reaching accuracies of up to 85.6%. Specifically in the human field, Jin et al. [32] have developed models capable of predicting macronutrient composition and detecting the risk of low milk production using metabolic fingerprints and ML, achieving an accuracy of 87.9%. These models capture texture patterns and density variations in the cream layer that are invisible to the human eye, consolidating artificial intelligence as an emerging standard in nutritional diagnosis.

The necessity for modernizing analytical procedures in the food industry has driven the adoption of efficient, non-invasive inspection technologies. As reviewed by Baiano [33], conventional techniques for liquid and semi-liquid products are often time-consuming and unsuitable for real-time monitoring. In this context, imaging-based systems have emerged as critical sensing modalities that provide spatial and multi-constituent information regarding the physicochemical properties of products like milk and oils. This transition towards vision-as-sensor frameworks is further supported by recent advancements in the dairy industry, where non-invasive computer vision methods have been successfully deployed to predict milk quality traits, such as fat and protein content, using affordable RGB camera systems [34]. These sensing architectures demonstrate that digitally extracted features from visible spectra can achieve high correlation coefficients with laboratory ground truths, effectively acting as non-contact optical transducers. Furthermore, recent reviews on food safety emphasize that the integration of AI with smart sensors and computer vision is shifting food quality assessment from slow laboratory testing to real-time, low-cost monitoring of liquid products [35]. While these imaging-based metrology systems have matured in agricultural and industrial sectors [36], their application in the clinical environment of neonatal units for human milk analysis remains an underexplored frontier. This study adapts these proven optical sensing principles to the high-precision requirements of neonatal nutritional assessment, where the imaging pipeline is calibrated to function as a high-precision physical gauge for biological samples.

Currently, there is no methodology that integrates low-cost hardware with a hierarchical segmentation process specifically designed to automate creatocrit in breast milk banks. The novelty of this work lies in the proposal of an objective metric derived from CVS that minimizes technical bias through geometric rectification and standardized optical conditioning, offering a robust and scalable alternative for resource-constrained environments.

## 3. Materials and Methods

**Figure 1:**
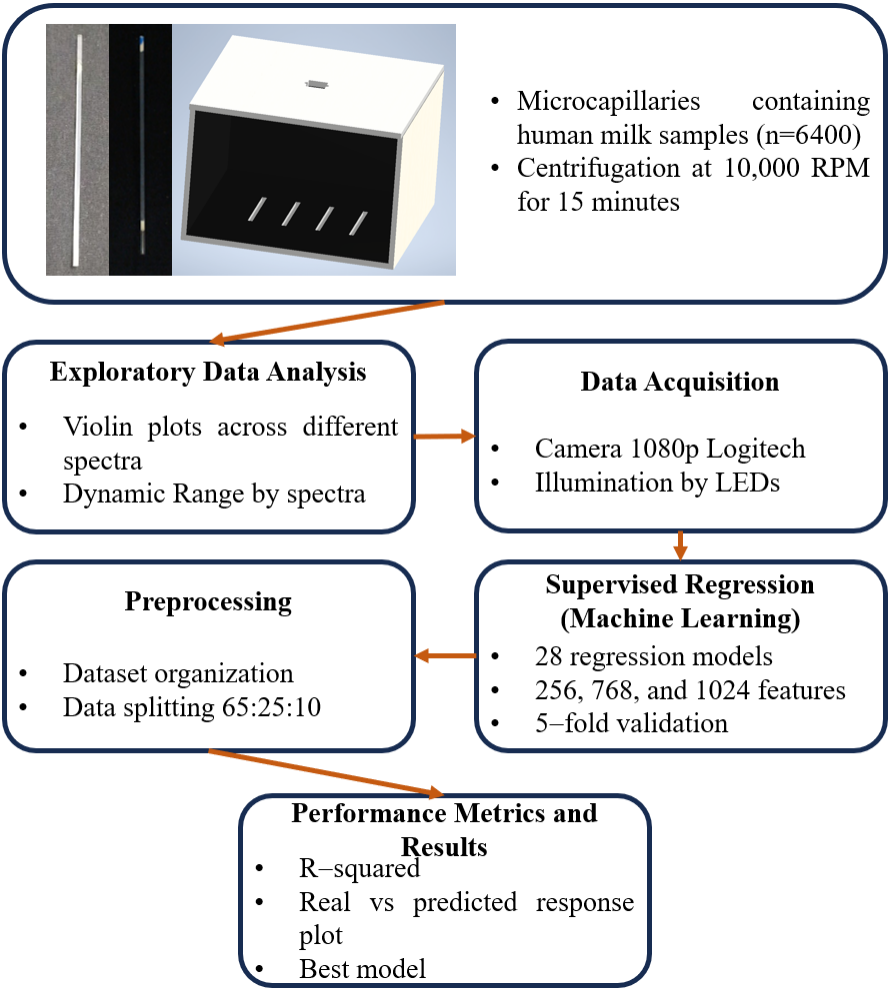
Detailed experimental scheme of the proposed Computer Vision System (CVS), illustrating the integrated workflow from biological sample preparation to automated digital quantification.

This section describes the integrated framework for automated lipid quantification, encompassing the physical experimental design, the hardware deployment of the Computer Vision System (CVS), and the mathematical modeling of measurement uncertainty. The study is structured to bridge the gap between traditional analog creamatocrit methods and automated digital estimation. As shown in Figure 1, the experimental scheme follows a sequential workflow: from the standardized preparation of biological samples to the high-resolution digital acquisition and subsequent predictive analysis.

### 3.1. Human Milk Samples and Experimental Design

A large-scale dataset of human milk samples (n=6400) was obtained from the Human Milk Bank of the San Antonio Abad del Cusco National Hospital (Cusco, Peru). The 6400 samples were obtained from a diverse donor pool during a standardized relabeling process at the regional hospital. In order to ensure data independence and maintain clinical anonymity, each individual milk unit was treated as a distinct biological unit. Furthermore, each milk unit corresponds to a single, unique digital image in the dataset. The experimental phase was structured to ensure metrological reliability through a standardized sample preparation and acquisition protocol. Individual samples were transferred to uniform glass microcapillaries (75 mm length, 1.1 mm internal diameter) and sealed with non-toxic plasticine. In order to achieve a clear physical separation between the lipid fraction and the serum, all microcapillaries underwent a centrifugation process at 10,000 RPM for 15 min using a dedicated clinical microcentrifuge. The study followed a randomized complete block design under controlled environmental conditions (22 ± 2 °C; 45–55% relative humidity). For the baseline analog measurement, a trained operator measured the cream height ($h_{c}$) and total height ($h_{t}$) using a precision manual caliper (1 mm resolution). Subsequently, for the digital workflow, the experimental setup was established within a controlled optical enclosure using low-intensity LEDs to minimize background noise. A high-resolution 1080p Logitech camera (Logitech, Lausanne, Switzerland) was positioned at the nadir view distance of 12 cm from the sample holder. In order to generate a robust training dataset, the vertical distances between the detected interfaces (seal–serum, serum–cream, and cream–seal) were computed for the digital images and individually corrected by a trained operator. These measurements act as a high-precision reference. The subsequent ML regression models were then developed to estimate the cream fraction (c) by analyzing global pixel intensity distributions, rather than relying solely on the boundary coordinates; this fraction is used to derive fat and energy quantification through established analytical equations.

### 3.2. Hardware Deployment and Optical Environment

**Figure 2:**
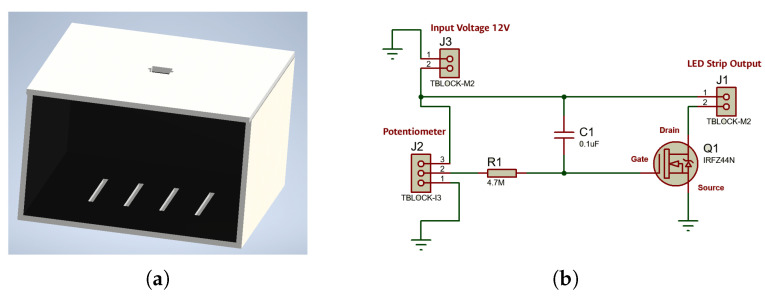
Lighting subsystem components and general assembly: (**a**) Chamber design; (**b**) Electronic control circuit.

CVS was integrated into a specialized optical enclosure designed to maintain consistency and eliminate environmental interference. The structure was manufactured in 5.5 mm High-Density Polyethylene. The design isolates the sample from ambient light interference. Its external dimensions are 13 cm (height) × 21 cm (length) × 22.5 cm (width) as shown in Figure 2a. In order to ensure optical axis stability, a mounting aperture is located at the top for a 1080p high-definition camera, Logitech StreamCam Plus (Logitech, Lausanne, Switzerland). This ensures the stability of the optical axis. The internal compartment is engineered for precise, repeatable placement of the microcapillary tube. The system uses constant-intensity LED strips (Philips, Amsterdam, The Netherlands). Golden light was established as the optimal light. A regulator with the IRFZ44N MOSFET (Infineon Technologies, Neubiberg, Germany) was implemented. The circuit operates at 12 V. A potentiometer allows manual brightness adjustment (see Figure 2b). The passive filtering stage eliminates noise.

**Figure 3:**
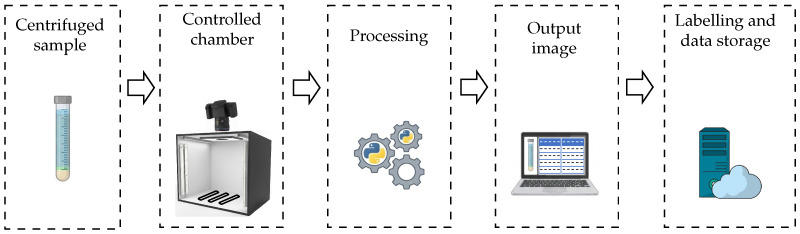
Workflow for image acquisition and processing.

A conditioning chamber with a black background and LED lighting was designed to ensure consistent optical conditions. Capture is performed with a 1080p Logitech camera connected via USB, selected for its precision in segmenting capillary layers. Edge computing is performed, and finally, the results are labelled and stored (see Figure 3).

#### 3.2.1. Light Sensitivity

**Figure 4:**
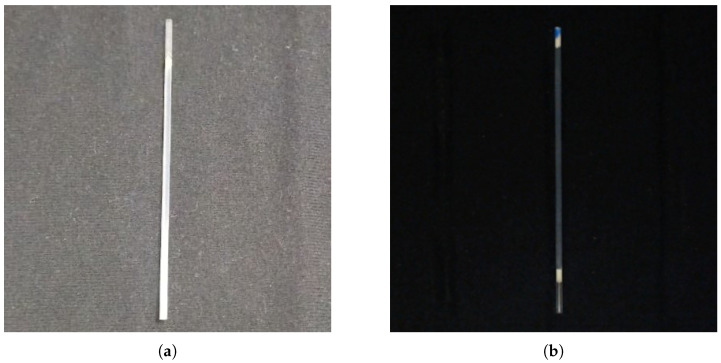
Impact of optical conditioning. Photo taken with (**a**) Ambient light and (**b**) in the chamber.

A series of experimental tests was conducted to optimize the edge definition of the microcapillary and maximize the visualization of its internal content. The parameters were locked at an exposure time of 10 ms, a gain of ISO 100, and a white balance of 4500 K, with the focus manually fixed at the center of the microcapillary axis. Initial evaluations confirmed that ambient light generates erratic reflections that compromise measurement repeatability. By comparing natural capture with the conditioned environment (Figure 4), the necessity of the black-background chamber for stable edge detection was validated.

**Figure 5:**
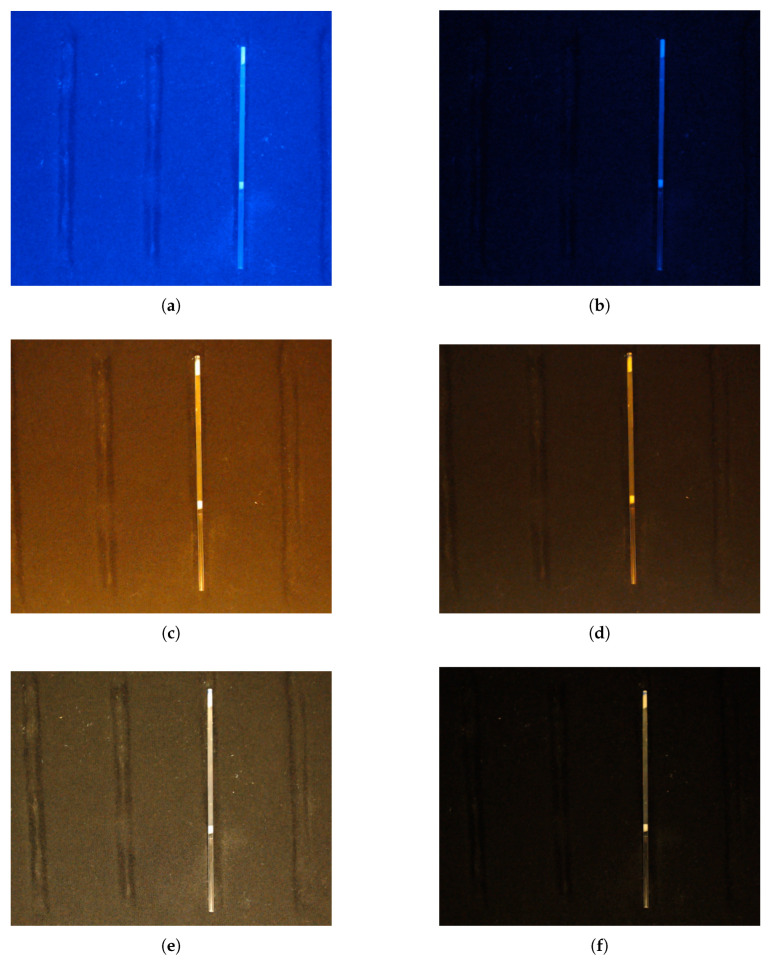
Comparison of microcapillaries under different light sources and levels. (**a**) Blue LED with high illumination. (**b**) Blue LED with low illumination. (**c**) Yellow LED with high illumination. (**d**) Yellow LED with low illumination. (**e**) Gold LED with high illumination. (**f**) Gold LED with low illumination.

Subsequently, the interaction between different light spectra and the microcapillary was evaluated using blue, yellow, and gold LED sources. As depicted in Figure 5, each spectrum was tested at high and low intensity levels to identify potential sensor saturation or low-contrast regions. To quantify these responses, a statistical analysis of pixel intensity distribution was performed using 30,000 random subsamples per configuration.

#### 3.2.2. Spectral Optimization

**Figure 6:**
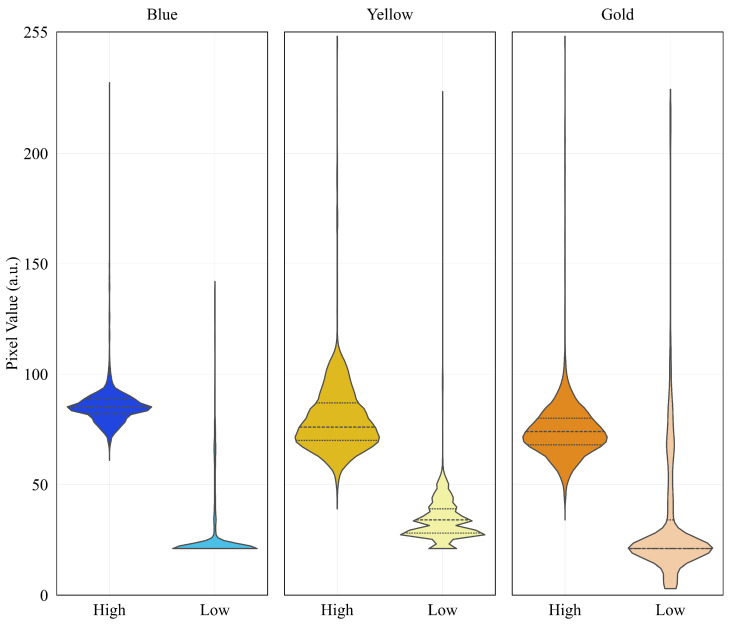
Distribution of pixel intensity (a.u.) across different spectra.

As summarized in Table 1 and visualized through the violin plots in Figure 6, the Gold Low spectrum demonstrated superior performance for artificial vision tasks. While blue and yellow sources exhibited higher noise floors or information loss at low levels, the Gold spectrum maintained a wide Dynamic Range (DR = 226 a.u.) and high homogeneity. Under these standardized conditions, the Dynamic Range (DR) was calculated as the intensity spread between the noise floor and the saturation limit across the RGB channels, reaching a peak of 226 a.u. for the Gold Low configuration. Specifically, the coincidence of $Q_{1}$ and $Q_{2}$ at 21.0 a.u. indicates a stable sensor response, while the minimum value of 3.0 a.u. facilitates a steeper intensity gradient. This enhanced contrast between the shadows and the microcapillary walls simplifies calibration of hierarchical segmentation algorithms, enabling robust distinction of physical boundaries regardless of minor ambient fluctuations.

### 3.3. Mathematical Framework and Uncertainty Analysis

The human milk fat percentage (*F*) and energy density (ED) are not measured directly but are derived from the cream layer height ($h_{c}$) and the total column height ($h_{t}$), which includes both the cream and serum layers. According to the standardized methodology established by [37], *F* and ED are calculated using the following Equations (1) and (2). These equations serve as the universal reference for international clinical protocols and technical health standards, ensuring consistency in the nutritional assessment of donor milk across global healthcare systems [38,39].

(1) $$F=\frac{2950}{73}\frac{h_{c}}{h_{t}}$$

(2) $$ED=6680\left(\frac{h_{c}}{h_{t}}\right)+290$$

In order to transform the Computer Vision System (CVS) from a simple image processing tool into a reliable metrological instrument, an uncertainty analysis was performed. This stage establishes the correspondence between the digital coordinate space (pixels) and the physical metric system, ensuring that the extracted heights possess physical meaning. By evaluating how resolution limits propagate through the analytical models, we can define the precision boundaries of the system.

#### 3.3.1. Error Propagation Model

In order to propagate the measurement error, a logarithmic transformation and subsequent differentiation were applied to Equation (1) to linearize the relationship between variables. Differentiating both sides yields the relationship between the relative uncertainties. Since uncertainties are additive in the worst-case scenario, the signs are taken as positive to determine the maximum possible absolute error (ΔF), resulting in Equation (3):

(3) $$\Delta F=F\left(\frac{\Delta h_{c}}{h_{c}}+\frac{\Delta h_{t}}{h_{t}}\right)$$

Likewise, for the energy density defined in Equation (2), the constant offset is subtracted before applying the logarithmic transformation and differentiation. The resulting absolute uncertainty for the ED is expressed in Equation (4):

(4) $$\Delta ED=\left(ED-290\right)\left(\frac{\Delta h_{c}}{h_{c}}+\frac{\Delta h_{t}}{h_{t}}\right)$$

#### 3.3.2. Mathematical Model for Uncertainty Analysis

By defining the cream fraction as $c=h_{c}/h_{t}$ and substituting the original definitions into Equations (3) and (4), we obtain the final numerical models in Equations (5) and (6).

(5) $$\Delta F=40.41\left(\frac{\Delta h}{h_{t}}\right)\left(1+c\right)$$

(6) $$\Delta ED=6680\left(\frac{\Delta h}{h_{t}}\right)\left(1+c\right)$$

The resulting expressions in Equations (5) and (6) reveal that the measurement uncertainty is governed by the cream fraction (*c*). Since the instrument resolution (Δh) constitutes a fixed constraint, defined either by the physical caliper with a nominal resolution of 1 mm (corresponding to an analog uncertainty of ±0.5 mm by definition of half-scale division) or by the digital pixel resolution (±1 px), and the total column height ($h_{t}$) remains constant for a standardized capillary volume, the propagation of error is effectively scaled by a constant coefficient. Consequently, the absolute uncertainty is minimal for samples with lower lipid content and increases linearly as the cream layer occupies a larger fraction of the tube. This mathematical behavior allows for a predictable assessment of sensor precision across the entire range of human milk compositions [40]. In order to fully characterize the digital measurement uncertainty beyond the discrete pixel resolution, three additional factors were addressed. First, lens distortion was minimized by using a fixed-focus assembly and maintaining a perpendicular optical axis relative to the microcapillary, ensuring a linear geometric projection within the Region of Interest (ROI) [41]. Regarding spatial calibration, the system operates directly in the raw pixel domain to maintain data integrity and avoid rounding errors associated with external metric conversions. Since the core variables are derived from the cream fraction (*c*), which is a dimensionless ratio, any physical scaling factor is mathematically cancelled, rendering the pixel-based measurement intrinsically self-calibrating as long as the sensor-to-sample distance remains constant. Finally, segmentation variability was mitigated through the standardized Gold Low illumination environment, which provides high-contrast interfaces and stable intensity gradients. This controlled setup ensures that the automated detection of $h_{c}$ and $h_{t}$ is repeatable and that the ±1 px resolution remains the dominant and predictable limit of the system uncertainty.

### 3.4. Computer Vision Pipeline

**Figure 7:**
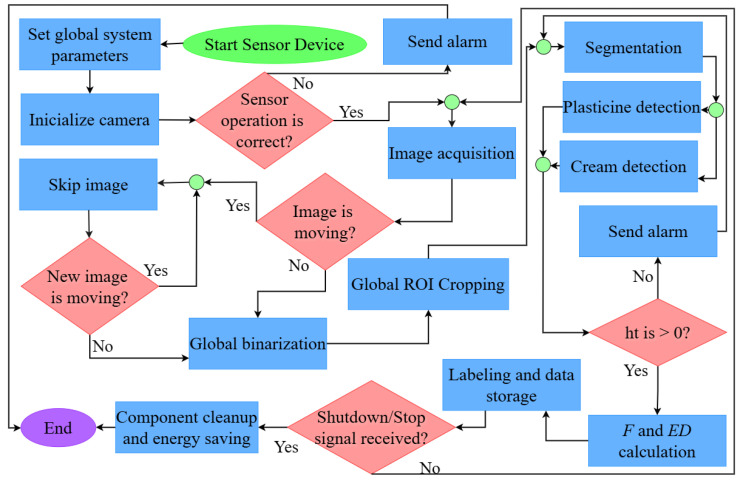
Proposed sensor operation algorithm.

The algorithm, developed in Python 3.10, processes images at their native resolution to avoid information loss from interpolation. The operational flow (see Figure 7) is based on segmenting raw units (pixels) to calculate the dimensions of the phases in the microcapillary.

A semiautomated labeling protocol was implemented to ensure the highest metrological standards during the training phase. While an initial segmentation algorithm was developed to estimate $h_{c}$ and $h_{t}$, each of the 6400 images captured underwent rigorous manual supervision and refinement. An expert reviewed and adjusted the detected boundaries in each sample to eliminate any residual errors caused by optical artifacts or meniscus distortion. This carefully selected dataset served as the high-precision ground truth (target). Consequently, the ML models were trained not only to replicate the initial segmentation but also to understand the complex relationship between global pixel distributions and achieve a level of robustness that surpasses simple automated edge detection.

#### 3.4.1. Hierarchical Segmentation and ROI Definition

From the original image $I\left(x,y\right)\in R^{M\times N\times 3}$, the conversion to greyscale $I_{g}\left(x,y\right)$ is performed. To isolate the microcapillary, global binarization is applied as described in Equation (7).

(7) $$B\left(x,y\right)=\left\{\begin{array}{cc} 255, & \mathrm{si}\;I_{g}\left(x,y\right)>T_{b},\\ 0, & \mathrm{si}\;I_{g}\left(x,y\right)\leq T_{b}, \end{array}\right.$$

where $T_{b}=20$ because it is sufficient to distinguish the microcapillary from the background. This value is determined empirically and standardized by Gold Low illumination environment. The contours ($C_{k}$) are extracted, and the geometry of the area (*A*) is validated using the shoelace formula derived from Green’s Theorem:

(8) $$A=\frac{1}{2}\left|\sum_{i=1}^{N}\left(x_{i}y_{i+1}-x_{i+1}y_{i}\right)\right|$$

On the other hand, the general Region of Interest (${\mathrm{ROI}}_{g}$) is defined by a bounding rectangle:

(9) $${\mathrm{ROI}}_{g}=\left\{\left(x,y\right)|x_{\min}\leq x\leq x_{\max},\;y_{\min}\leq y\leq y_{\max}\right\}.$$

The system divides the ${\mathrm{ROI}}_{g}$ using the midpoint $y_{mid}=\left\lfloor h/2\right\rfloor$. An intensity threshold $T_{w}=90$ is applied to isolate the seal (${\mathrm{ROI}}_{1}$) and the cream (${\mathrm{ROI}}_{2}$). The lower boundary of the seal ($y_{seal}$) is defined as:

(10) $$y_{seal\_end}=y_{base}+\Delta y_{seal}+h_{seal}$$

where $y_{base}$ is the origin of the capillary, $\Delta y_{seal}$ is the relative displacement, and $h_{s}$ is the height of the seal. The upper limit of the cream ($y_{c\_start}$) is located in the second subregion as described in Equation (11) where $\Delta y_{c}$ is the relative displacement of the cream:

(11) $$y_{c\_start}=y_{base}+y_{mid}+\Delta y_{c}$$

where $\Delta y_{c}$ represents the displacement of the cream layer relative to the start of the second subregion. This spatial discrimination allows the analysis of the serum phase to be confined to a third region of interest (${\mathrm{ROI}}_{3}$) defined by Equation (12).

(12) $$ROI_{3}=\left\{\left(x^{\prime \prime },y^{\prime \prime }\right)|x_{min}\leq x^{\prime \prime }\leq x_{max},\;y_{seal\_end}+1\leq y^{\prime \prime }\leq y_{c\_start}-1\right\}$$

A grey range $\left[T_{\min}^{g}=25,T_{\max}^{g}=90\right]$ is applied to extract the dominant contour of the serum. The final heights of the serum ($h_{s}$) and cream ($h_{c}$) in pixels are obtained by adjusting a rectangle of minimum area:

(13) $$h_{px}=\max\left(w,h\right)$$

where *w* is the width and *h* is the height. Finally, based on these values, the percentage of cream, the percentage of *F*, and the total ED are calculated using analytical expressions aligned with the technical guidelines established in [39] and based on the formulations proposed by [37].

#### 3.4.2. Adaptive Cropping

To ensure that color analysis is restricted to the biological content of the sample, an adaptive cropping algorithm is implemented. The adaptive cropping is performed only along the vertical axis (*y*). Once the general Region of Interest (${\mathrm{ROI}}_{g}$) has been defined, the final vertical limits of the sample (see Equations (14) and (15)) are computed by detecting intensity transitions corresponding to the lower boundary of the seal and the lower boundary of the cream layer, respectively.

(14) $$y_{seal\_end}=y_{base}+{arg max}_{y\in {\mathrm{ROI}}_{1}}\left\{\nabla I_{g}\left(y\right)>T_{w}\right\}+h_{seal}$$

(15) $$y_{cream\_end}=y_{c\_start}+{arg max}_{y\in {\mathrm{ROI}}_{2}}\left\{\nabla I_{g}\left(y\right)>T_{w}\right\}+h_{cream}$$

where $\nabla I_{g}$ denotes the vertical intensity gradient. No additional offset is applied; therefore, the cropping limits coincide with the detected physical interfaces. The resulting cropped image $I_{crop}$ is defined as the subset of pixels containing exclusively the serum and cream phases, effectively removing external artefacts and non-biological regions.

The horizontal extent of the crop is defined by the full width of the microcapillary ($x_{\min}$ to $x_{\max}$), ensuring that all columns of the sample are included in the analysis.

#### 3.4.3. Multichannel Statistical Feature Extraction

After isolating the sample, intensity-domain feature extraction is performed to feed the regression models. For each cropped image $I_{crop}$, the RGB color channels are separated, and the luminance (Gray Scale) channel is computed. The feature vector *X* is constructed by computing frequency histograms for each channel k∈{R,G,B,Gray}, as shown in Equation (16).

(16) $$H_{k}\left(i\right)=\sum_{x,y}\delta \left(I_{crop,k}\left(x,y\right)-i\right),\;i\in \left[0,255\right],$$

where δ is the Kronecker delta function, and *i* represents the intensity level. The final feature space is defined by concatenating these histograms. Using this procedure, a structured dataset of feature vectors was generated, where each row corresponds to a single sample image and each column represents a specific feature. This organized dataset was then ready for the application of ML algorithms for regression and predictive analysis.

### 3.5. Predictive Modeling via ML Regression Algorithms

In order to identify the algorithm with the highest predictive capacity for estimating the cream fraction (*c*), 28 ML regression models were evaluated. These models were grouped into eight main families: linear models (EL), ensemble trees (ESB), Gaussian process regressors (GPR), kernel-based methods (KN/SVM), linear regression models (LR), neural networks (NN), and decision trees (Tree). Table 2 provides the specific configurations and hyperparameters used for each architecture. The total set of images was divided into 65% for training, 25% for validation, and the remaining 10% for testing. Each model was evaluated under identical computational conditions. The selection criterion for the optimal model was based on maximizing the coefficient of determination ($R^{2}$).

## 4. Results

**Figure 8:**
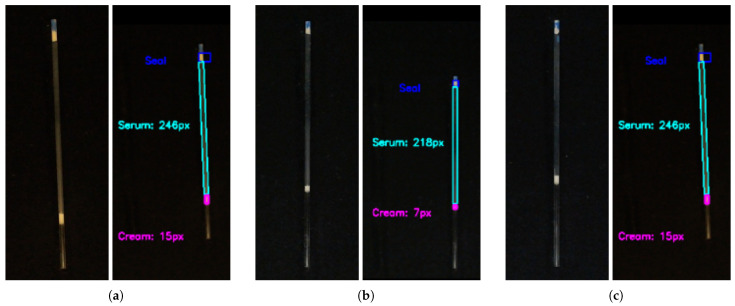
Automatic segmentation under diverse conditions. (**a**) Inclined sample. (**b**) Vertical alignment. (**c**) Incomplete filling.

This section presents the performance of the developed computer vision system (CVS). The system analyzed images of microcapillaries with centrifuged human milk samples. The algorithm automatically segmented the cream and serum layers. Figure 8 shows the system’s performance under different experimental conditions.

### 4.1. Segmentation Robustness Analysis

The system proved robust against physical variations during acquisition. Sample 1 (Figure 8a) shows a slight axial tilt. The algorithm correctly segmented the phases despite this deviation. Sample 2 (Figure 8b) demonstrates an ideal vertical alignment. Sample 3 (Figure 8c) shows incomplete filling of the capillary. In all cases, the CVS correctly identified the critical fluid boundaries. Regarding the cream fraction (*c*), we yield a Mean Absolute Error (MAE) of 0.0050 and a Root Mean Square Error (RMSE) of 0.0062. To further assess the discrepancy between methods, the digital sensor was established as the high-precision reference—constrained only by a fixed spatial resolution of 1 px—revealing that manual measurements exhibited a Mean Relative Error (MRE) of 9.52%. A paired *t*-test confirmed no statistically significant difference between the digital and manual methods (*p* = 0.572), with a negligible mean bias of 0.06%. This high degree of agreement between the digital and manual methods, characterized by the absence of systematic bias, confirms the clinical relevance of the CVS as a high-precision alternative to traditional crematocrit scales.

### 4.2. Uncertainty Analysis

**Figure 9:**
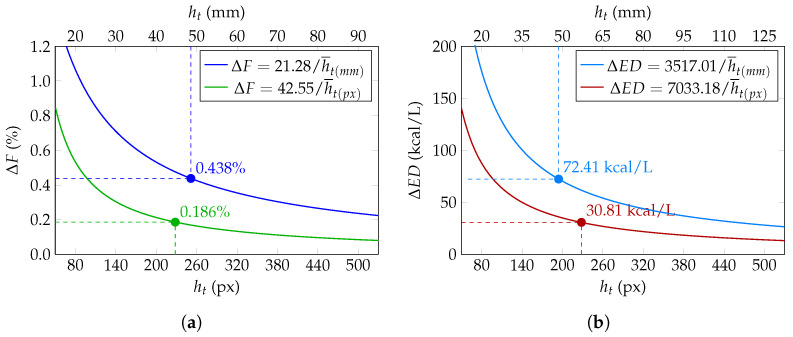
Comparison of uncertainty for: (**a**) Fat content and (**b**) Energy Density. For ΔF, blue and green curves represent analog and CVS measurement, respectively. For ΔED, light blue and red curves denote analog and CVS quantification.

Uncertainty was evaluated under real-world conditions. The study included 68 total measurements for validation. While the ML models were trained and validated using the full dataset (n = 6400), the uncertainty analysis and its graphical representation were conducted on a representative subset of 34 samples, selected across the full concentration range to ensure visual clarity and avoid over-plotting. This setup ensures a balanced comparison between both techniques. Experimental mean values were: ${\bar{c}}_{\left(mm/mm\right)}=0.053$, ${\bar{c}}_{\left(px/px\right)}=0.0529$, ${\bar{h}}_{t(mm)}=48.57$ mm and ${\bar{h}}_{t(px)}=228.20$ px. These values were substituted into Equations (5) and (6). The comparative analysis of measurement uncertainties is presented in Figure 9. For fat content, Figure 9a shows that conventional analog quantification results in an uncertainty of ±0.438%, whereas the proposed CVS reduces this value to ±0.186%. Similarly, for energy density, Figure 9b illustrates a reduction from ±72.41 kcal/L using analog methods to ±30.81 kcal/L with the CVS.

The statistical impact of this improvement is quantified in Table 3. For the mean fat content ($\bar{F}\approx 2.01\%$), the analog relative error of 21.21% was reduced to 9.00% using the digital approach. Similarly, the mean energy density ($\bar{ED}\approx 622.49$ kcal/L) showed a reduction from 11.52% to 4.90%. These results confirm a relative error improvement of approximately 57.5%, validating the CVS as a robust tool for high-fidelity nutrient estimation.

**Figure 10:**
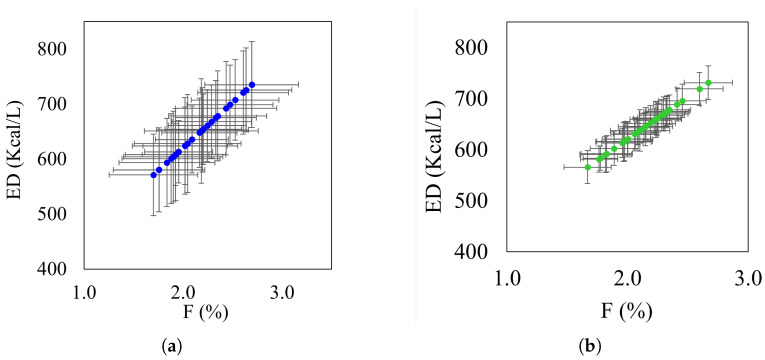
Uncertainty intervals for: (**a**) analog and (**b**) digital measurement.

Since both *F* and ED depend on $h_{c}$ and $h_{t}$, their linear correlation is expected. Figure 10 illustrates this relationship, where Figure 10a reveals the high uncertainty inherent in analog measurements, characterized by wide error intervals across the experimental range. In contrast, Figure 10b demonstrates a drastic reduction in uncertainty through the CVS system, which presents significantly narrower and more precise intervals.

### 4.3. Performance Analysis of Regression Models

**Figure 11:**
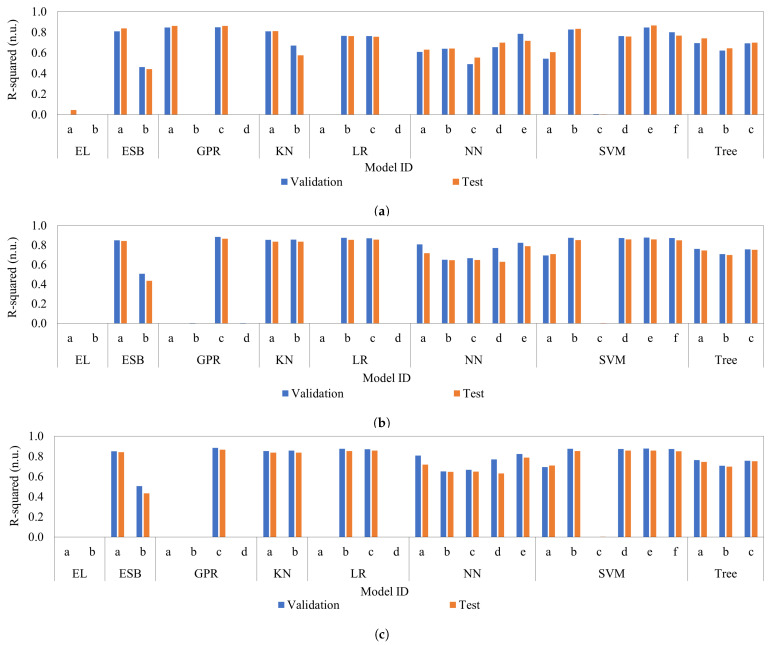
Performance of ML Algorithms for Estimate Cream Fraction (*c*) for: (**a**) Gray Scale, (**b**) RGB, and (**c**) RGB + Gray Scale.

Figure 11 illustrates the $R^{2}$ values achieved using Gray Scale (Figure 11a), RGB (Figure 11b), and combined RGB + Gray Scale (Figure 11c) descriptors with 256, 768, and 1024 features, respectively. The validation phase is represented in blue, while the testing phase is shown in orange. All models are identified by their specific ID and categorized according to the parameters detailed in Table 2.

**Figure 12:**
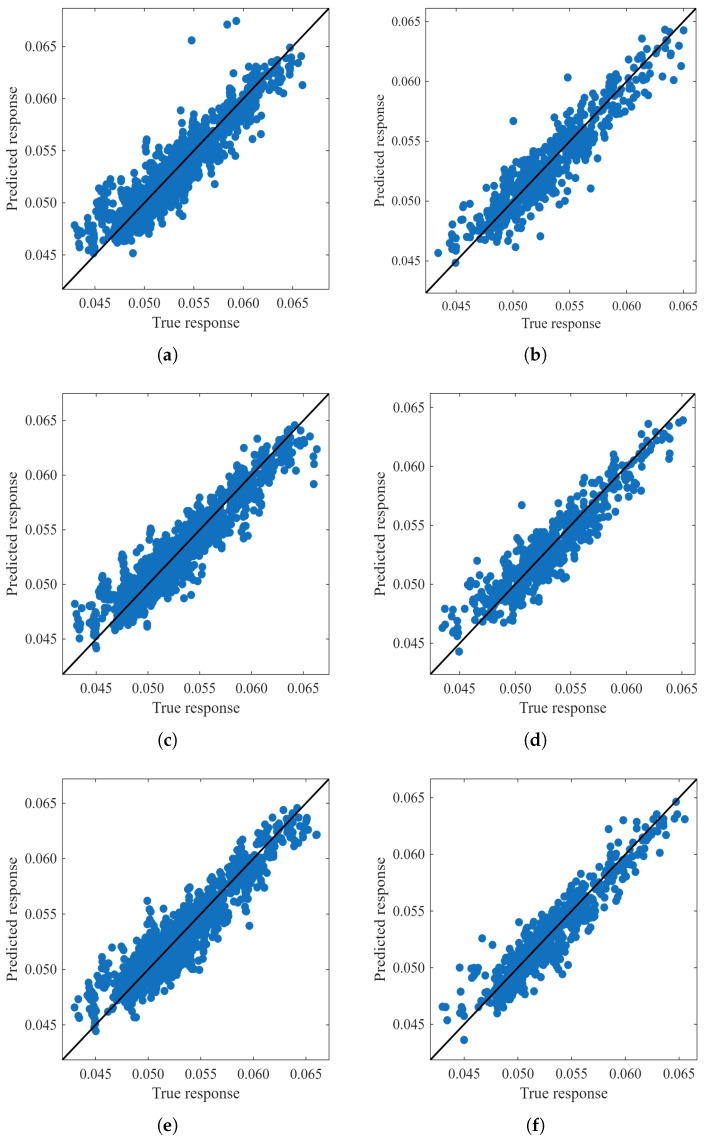
Comparison of Regression Models for Cream Fraction (*c*) estimation using different feature sets: Gray Scale for (**a**) Validation and (**b**) Test. RGB for (**c**) Validation and (**d**) Test. RGB + Gray Scale for (**e**) Validation and (**f**) Test.

For the 256 Gray Scale features, the four best models were: Rational Quadratic GPR (GPR c) with $R^{2}$ of 0.850 for validation (see Figure 12a) and 0.862 for test (see Figure 12b), Exponential GPR (GPR a) with 0.848 and 0.863, Medium Gaussian SVM (SVM e) with 0.847 and 0.866, and Cubic SVM (SVM b) with 0.827 and 0.834. These results demonstrate that even with limited intensity data, GPR and SVM architectures maintain high stability.

In the 768 RGB configuration, performance increased significantly. The top four models were: Rational Quadratic GPR (GPR c) reaching an $R^{2}$ of 0.885 for validation (see Figure 12c) and 0.867 for test (see Figure 12d), followed by Medium Gaussian SVM (SVM e) with 0.878 and 0.859, Linear Regression (LR b) with 0.876 and 0.854, and Cubic SVM (SVM b) with 0.876 and 0.854. The inclusion of color channels improved the model’s ability to distinguish cream fraction-related chromatic patterns.

Finally, for the 1024 RGB + Gray Scale features, the models showed the best generalization. The leading models were: Rational Quadratic GPR (GPR c) with $R^{2}$ of 0.870 for validation (see Figure 12e) and 0.871 for test (see Figure 12f), Medium Gaussian SVM (SVM e) with 0.864 and 0.878, Cubic SVM (SVM b) with 0.862 and 0.856, and Quadratic SVM (SVM f) with 0.859 and 0.846. The combination of 1024 descriptors minimized the variance between phases, proving that integrating luminance with color data yields the most reliable quantification.

These findings highlight the ability of the sensor to provide high-quality data for regression modeling. The 768 RGB feature set proved to be the most accurate, achieving a peak test $R^{2}$ of 0.867 with the Rational Quadratic GPR model. This performance confirms that chromatic signatures are the most decisive descriptors for quantifying the cream fraction. Furthermore, selecting this configuration ensures that the system maintains a better $R^{2}$ with a resolution of ±1 pixel, effectively converting high-dimensional image data into a stable clinical measurement with a 57.5% reduction in final uncertainty.

### 4.4. Feature Importance Analysis

**Figure 13:**
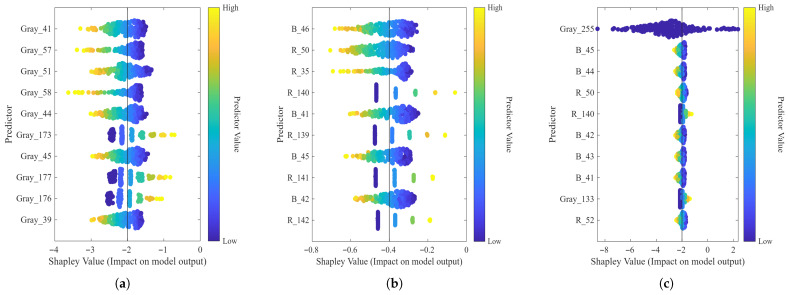
Feature importance analysis using SHAP values ($\times 10^{-3}$) for: (**a**) Grayscale, (**b**) RGB, and (**c**) combined RGB + Gray Scale features.

A feature importance analysis was performed using SHAP (Shapley Additive exPlanations) values. Figure 13 illustrates the importance distribution of the 10 best features across the Gray Scale, RGB, and RGB + Gray Scale.

In the Gray Scale configuration (see Figure 13a), the model shows a high statistical dependency on low-to-mid intensity bins to establish a negative baseline for the prediction. Predictors such as Gray_41, Gray_57, Gray_51, and Gray_58 show that high feature values result in more negative SHAP values, which the model statistically associates with non-lipid regions such as the background or serum phase. Conversely, features like Gray_173 and Gray_177 display a different behavior, where higher intensity values correlate with a positive SHAP impact, suggesting these bins are significant predictors for the cream layer within the learned feature space.

The RGB analysis (see Figure 13b) reveals a complex spectral interaction, where the Blue (B) and Red (R) channels provide the most discriminative data. The predictor B_46 is the most significant; however, its high values correlate with more negative SHAP impacts, suggesting it serves as a statistical boundary marker. A relevant finding is the role of red channel features like R_140 and R_141, where high intensity values result in positive SHAP shifts. This suggests a statistical correlation between these specific spectral bins and the target variable, rather than a causal physical interaction.

In the 1024-features combined space (Figure 13c), the model’s behavior shifts towards a dominant statistical weight on luminance saturation. The feature Gray_255 emerges as the most significant predictor by a wide margin. This statistical association suggests that the model prioritizes maximum brightness values to differentiate the cream layer. By assigning high importance to Gray_255, the regression model effectively minimizes the impact of mid-tone and shadow regions, focusing the mathematical prediction on peak intensity data rather than a direct physical modeling of lipid scattering mechanisms.

## 5. Discussion

The transition from manual inspection to the proposed CVS represents a fundamental shift in measurement principles, as synthesized in the cross-method evaluation in Table 4. While electrochemical sensors [13,16] rely on ion-selective redox reactions—which offer high chemical sensitivity but are susceptible to thermal noise and electrode fouling—the CVS employs a non-invasive optical reflectance-based methodology. Unlike electrochemical methods that require periodic reagent calibration, the CVS maintains long-term stability through fixed hardware optimization. The 57.5% relative error improvement should be interpreted as a meteorological milestone in interface detection, directly resulting from the peak performance of the Rational Quadratic GPR model using the 768 RGB feature set ($R^{2}=0.867$). This model has a training time of 573.4 s, a prediction Speed of 2807.9 obs/s, and a model size of 33.9 MB. The computational feasibility was validated on a portable workstation with an Intel Core i7-1255U processor and 12GB of RAM, where the total processing time per sample remained consistently under 1.5 s.

In manual clinical practice, factors such as the parallax effect and subjective boundary identification lead to an MRE of 9.52%. The CVS eliminates this variance by applying a probabilistic sub-pixel analysis to the chromatic signatures of the milk phases. This improvement is not merely a statistical gain but a reduction in the noise floor of the Creamatocrit method, effectively shifting the measurement from a human-centered estimation to a standardized digital quantification. The superior performance of the Rational Quadratic GPR model is rooted in its kernel structure, which is uniquely suited for the physical nature of milk samples. Unlike linear kernels or the rigid architectures of CNNs [31] that may overfit small clinical datasets, the Rational Quadratic kernel acts as a scale mixture of RBF kernels. This allows the model to handle both small-scale chromatic fluctuations and larger-scale trends in the c fraction simultaneously.

The proposed CVS offers a low-cost, automated alternative for Human Milk Banks in resource-limited settings and low-income countries. By replacing manual inspection with a digital scan, the system eliminates inter-operator variability and parallax errors. This allows non-specialized clinical staff to perform precise, real-time nutritional fortification for infants, providing a scalable, standardized tool where expensive infrared analyzers are not feasible.

## 6. Conclusions

This research successfully developed an optimized CVS for the automated estimation of the cream fraction, which is subsequently used to calculate the quantification of fat and energy in human milk through established analytical equations. The integration of hardware-level optimization and ML demonstrates a measurable advancement in metrological reliability for neonatal care, rather than a mere incremental improvement. This progress is characterized by two distinct technical contributions: first, the hardware optimization and illumination control effectively lowered the measurement noise floor, enabling a 57.5% reduction in final uncertainty; second, the Rational Quadratic GPR model provided the necessary sub-pixel precision and stability ($R^{2}=0.867$) to maintain a negligible bias of 0.06% across the clinical range.

Despite these advancements, certain limitations must be acknowledged. While the CVS eliminates analog errors such as parallax, its performance remains dependent on the physical quality of the capillary filling and a controlled acquisition environment. Furthermore, the current validation is focused on a specific clinical range, with observed cream fraction values between 0.0429 and 0.0663, which correspond to energy density levels between 576.8 and 732.9 kcal/L. These values correspond to the typical normative range for mature human milk; consequently, further studies are required to ensure the system’s generalization across extreme concentrations, such as those found in colostrum (potentially higher) or in cases of severe maternal malnutrition (potentially lower).

Future work will focus on two key areas. First, we will prioritize scalability and mobile integration by porting the CVS to smartphone platforms [28], enabling home-based nutritional monitoring for lactating individuals. Second, the target biomarker database will be expanded to include the quantification of total proteins and lactose levels [13]. In order to ensure model robustness under real-world conditions, future validation will include non-normative milk samples, considering diverse maternal profiles, seasonal variations, and extreme fat concentrations. Additionally, multimodal sensor fusion and advanced preprocessing techniques will be explored [42] to isolate biological responses from environmental effects. These advancements will not only enhance individual neonatal care but also provide a scalable tool for large-scale epidemiological surveillance of maternal-infant nutritional status, ensuring global clinical applicability even in the most remote regions.

## Figures and Tables

**Table 1:** Statistical distribution of pixel value (a.u.).

Color	Level	Q1	Q2	Q3	Min	Max	DR ^1^
Blue	High	82.0	85.0	89.0	61	232	171
	Low	21.0	21.0	21.0	21	142	121
Gold ^2^	High	68.0	74.0	80.0	34	253	219
	**Low**	**21.0**	**21.0**	**34.0**	**3**	**229**	**226**
Yellow	High	70.0	76.0	87.0	39	253	214
	Low	28.0	34.0	39.0	21	228	207

^1^ DR is Dinamic Range. ^2^ Bold values indicate the optimal performance of the Gold spectrum.

**Table 2:** ML models, presets, and hyperparameter configurations used in the analysis.

Model ID	Preset	Hyperparameters
EL a	Efficient Linear Least Squares	Learner: Least squares
EL b	Efficient Linear SVM	Learner: SVM
ESB a	Bagged Trees	Minimum leaf size: 8
ESB b	Boosted Trees	Minimum leaf size: 8
GPR a	Exponential GPR	Basis function: Constant
GPR b	Matern 5/2 GPR	Basis function: Constant
GPR c	Rational Quadratic GPR	Basis function: Constant
GPR d	Squared Exponential GPR	Basis function: Constant
KN a	Least Squares Regression Kernel	Learner: Least Squares Kernel
KN b	SVM Kernel	Learner: SVM
LR a	Interactions Linear	Terms: Interactions
LR b	Linear	Terms: Linear
LR c	Robust Linear	Terms: Linear
LR d	Stepwise Linear	Initial terms: Linear
NN a	Bilayered NN	N° of fully connected layers: 2
NN b	Medium NN	N° of fully connected layers: 1
NN c	Narrow NN	N° of fully connected layers: 1
NN d	Trilayered NN	N° of fully connected layers: 3
NN e	Wide NN	N° of fully connected layers: 1
SVM a	Coarse Gaussian SVM	Kernel function: Gaussian
SVM b	Cubic SVM	Kernel function: Cubic
SVM c	Fine Gaussian SVM	Kernel function: Gaussian
SVM d	Linear SVM	Kernel function: Linear
SVM e	Medium Gaussian SVM	Kernel function: Gaussian
SVM f	Quadratic SVM	Kernel function: Quadratic
Tree a	Coarse Tree	Minimum leaf size: 36
Tree b	Fine Tree	Minimum leaf size: 4
Tree c	Medium Tree	Minimum leaf size: 12

**Table 3:** Comparison of Relative Errors between Analog Error and Digital Error Measurement.

Parameter	Analog Error (%)	Digital Error (%)	R.E.I. (%) ^1^
*F*	21.21	9.00	57.6
ED	11.52	4.90	57.5

^1^ R.E.I. is the Relative Error Improvement, representing the percentage reduction in the relative error achieved by the gold spectrum CVS compared to manual quantification.

**Table 4:** Comparison of methodologies in recent milk and food analysis studies.

Authors	Methodology	Task	Target	Metric	Key Result
[16]	Electrochemical	Monit.	Nutrients	Access.	Biomarker
[13]	Electrochemicals	Quant.	Glucose	Accuracy	96.8–104.1%
[31]	CNN	Classif.	Cow yield	Accuracy	85.64%
[32]	G.B.	Predict.	Production	Accuracy	87.9%
This Work	CVS + ML	Quant.	Fat/Energy	$R^{2}$	0.867

Note: Quant. = Quantification; Monit. = Monitoring; G.B. = Gradient Boosting; Predict. = Prediction; Classif. = Classification; Access. = Accessibility; R.E.I. = Relative Error Improvement.

## Data Availability

The data provided can be found within the article. The original contributions made in this study are included in the document; any additional inquiries can be directed to the author or authors responsible.
